# Highly conductive V_4_C_3_T_*x*_ MXene-enhanced polyvinyl alcohol hydrogel electrolytes for flexible all-solid-state supercapacitors

**DOI:** 10.3389/fchem.2024.1482072

**Published:** 2024-10-09

**Authors:** Xiaoqing Bin, Minhao Sheng, Wenxiu Que

**Affiliations:** Electronic Materials Research Laboratory, Key Laboratory of the Ministry of Education, International Center for Dielectric Research, Shaanxi Engineering Research Center of Advanced Energy Materials and Devices, School of Electronic Science and Engineering, Xi’an Jiaotong University, Xi’an, China

**Keywords:** V4C3Tx MXene, hydrogel electrolyte, self-healing, flexible device, all-solid-state supercapacitor

## Abstract

Hydrogel electrolytes are an integral part of flexible solid-state supercapacitors. To further improve the low ionic conductivity, large interfacial resistance and poor cycling stability for hydrogel electrolytes, the V_4_C_3_T_*x*_ MXene-enhanced polyvinyl alcohol hydrogel electrolyte was fabricated to enhance its mechanical and electrochemical performance. The high-conductivity V_4_C_3_T_*x*_ MXene (16,465.3 S m^−1^) bonding transport network was embedded into the PVA-H_2_SO_4_ hydrogel electrolyte (PVA- H_2_SO_4_-V_4_C_3_T_*x*_ MXene). Results indicate that compared to the pure PVA-H_2_SO_4_ hydrogel electrolyte (105.3 mS cm^−1^, 48.4%@2,800 cycles), the optimal PVA-H_2_SO_4_-V_4_C_3_T_*x*_ MXene hydrogel electrolyte demonstrates high ionic conductivity (133.3 mS cm^−1^) and commendable long-cycle stability for the flexible solid-state supercapacitors (99.4%@5,500 cycles), as well as favorable mechanical flexibility and self-healing capability. Besides, the electrode of the flexible solid-state supercapacitor with the optimal PVA-H_2_SO_4_-V_4_C_3_T_*x*_ MXene hydrogel as the solid-state electrolyte has a capacitance of 370 F g^−1^ with almost no degradation in capacitance even under bending from 0° to 180°. The corresponding energy density for flexible device is 4.6 Wh kg^−1^, which is twice for that of PVA-H_2_SO_4_ hydrogel as the solid-state electrolyte.

## 1 Introduction

As the proliferation of portable electronics, wearable technology, smart homes, and Internet of Things devices continues, there is a rising demand for lightweight, flexible, and high-efficiency energy storage solutions (Anasori et al., 2017; Wei et al., 2024; Bin et al., 2022; Feng et al., 2023). Flexible solid-state supercapacitors are well-suited to address these demands. Hydrogel electrolytes are an integral part of flexible solid-state devices, they are usually composed of a polymer matrix and a liquid electrolyte, where a large amount of liquid electrolyte is absorbed into the polymer matrix to form a gel-like three-dimensional network structure (Boota and Gogotsi, 2018; Chang et al., 2018; Zhang et al., 2018; Zhang et al., 2017). This unique structure possesses the stability of solid electrolytes while maintaining the high ionic conductivity of liquid electrolytes. Currently studied polymer gel electrolyte matrices generally include potassium polyacrylate (PAAK), polyacrylamide (PAAM), polyvinyl alcohol (PVA), and polyvinyl alcohol/polyacrylic acid (PVA/PAA) hydrogels (Dong et al., 2018; Yu et al., 2018; Luo et al., 2022; Pang et al., 2018). Among these, polyvinyl alcohol (PVA) is more attractive due to its biodegradability, non-toxicity, low cost, and chemical stability. PVA-H_2_SO_4_ is often used as the electrolyte for flexible solid-state supercapacitors (VahidMohammadi et al., 2021; Sheng et al., 2021). On one hand, the addition of H_2_SO_4_ provides a proton source for the hydrogel electrolyte, on the other hand, it has a terrible effect on the mechanical properties for the hydrogel, disrupting the intermolecular hydrogen bonds between PVA chains and water molecules (Guo et al., 2018; Zhu et al., 2024). Besides, compared to liquid electrolytes, the low ionic conductivity, large interfacial resistance and poor cycling stability of hydrogel electrolytes limit the further development of flexible solid-state devices (Lukatskaya et al., 2017).

MXene’s unique electronic structure, tunable interlayer spacing, and abundant functional groups have made it shine in the field of energy storage (Bin et al., 2021; Bin et al., 2023). It exhibits a two-dimensional layered structure, and the general formula of MXene is M_n+1_X_n_T_*x*_, where M represents a transition metal, X is carbon or nitrogen, and T_*x*_ refers to surface groups (such as -I, -OH, -Cl, -O, -Br, -OH, -S, -F, etc.) (Li et al., 2023; Jee et al., 2024). Due to the diversity of transition metals and surface functional groups, the properties of MXene can be tuned by selecting combinations of transition metals and X elements, as well as by controlling their surface chemical groups (Sheng et al., 2022; Bin et al., 2024). The two-dimensional, layered structure of V_4_C_3_T_*x*_ MXene nanosheets naturally forms channels to facilitate ion transport, making them ideal for charge storage applications. V_4_C_3_T_*x*_ MXene, which has the large interlayer spacing (2.1 nm) and the outstanding electrical conductivity (16,465.3 S m^−1^), exhibits remarkable rate performance and the splendid cycle stability (Bin et al., 2022; Bin et al., 2021). The outer layer of transition metals element vanadium with variable valence state can provide abundant redox sites, which conform to the pseudocapacitive energy storage characteristics, while the inner layer of transition metal carbides rapidly transports electrons. Additionally, two-dimensional V_4_C_3_T_*x*_ MXene also possesses good mechanical flexibility, holding great potential for the application of flexible supercapacitor devices. Introducing highly conductive V_4_C_3_T_*x*_ MXene as an inorganic filler in organic hydrogel electrolytes is an effective strategy to enhance its ionic conductivity. Xu et al. designed a three-dimensional coaxial confined MXene-based solid polymer electrolyte by introducing Ti_3_C_2_T_*x*_ MXene nanosheets into polyacrylonitrile (PAN) fibers, exhibiting excellent ionic conductivity (3.07 × 10^−3^ S cm^−1^) (Xu et al., 2024). Chen et al. prepared a solid-state electrolyte using an inorganic compatibilizer, polyacrylonitrile grafted MXene (MXene-g-PAN), and the constructed energy storage device had a capacity of 131 mAh g^−1^ (Chen et al., 2023). Feng et al. proposed a design concept of a multifunctional MXene-bonded transport network embedded in a solid-state electrolyte to achieve efficient and uniform ion transport within the electrolyte, which could improve the mechanical properties and thermal diffusion performance for the solid-state electrolyte (Feng et al., 2022).

Therefore, the V_4_C_3_T_*x*_ MXene-enhanced polyvinyl alcohol hydrogel electrolytes were designed to increase the ionic conductivity, minimize interfacial resistance for hydrogel electrolytes, and further enhance its mechanical and electrochemical performance. The high-conductivity V_4_C_3_T_*x*_ MXene (16,465.3 S m^−1^) bonding transport network was embedded into the PVA-H_2_SO_4_ hydrogel electrolyte, which was prepared by cyclic freeze-thawing for physical cross-linking coupled with a mechanical blending method. The enhancement mechanism of V_4_C_3_T_*x*_ MXene on polyvinyl alcohol containing sulfuric acid (PVA-H_2_SO_4_) may involve the following aspects. First of all, the surface of MXene typically contains rich functional groups, such as hydroxyl (-OH), to form hydrogen bonds with the hydroxyl groups on PVA molecules. The formation of hydrogen bonds helps to improve the interfacial compatibility and overall stability for the hydrogel electrolyte. Furthermore, the interaction between sulfate ions (SO_4_^2−^) and the metal sites on the MXene surface, resulting in the formation of ionic bonds, significantly boosts the ionic conductivity of the electrolyte. Additionally, there may be van der Waals forces between the MXene nanosheets and PVA molecules, which help to maintain the structural stability for the hydrogel electrolyte. Thus, the V_4_C_3_T_*x*_ MXene/polyvinyl alcohol hydrogel (PVA) electrolyte has significant advantages in increasing the ionic conductivity for hydrogel electrolytes, improving interfacial contact, and enhancing mechanical and electrochemical properties, which is of great importance for the development of flexible solid-state electrolytes with high-performance.

## 2 Experimental

### 2.1 Preparation of V_4_C_3_T_*x*_ MXene/polyvinyl alcohol (PVA) hydrogel electrolytes

Firstly, the V_4_C_3_T_*x*_ MXene was synthesized as reported in our previous work. Typically, the precursor V_4_AlC_3_ (MAX) powder with a molar ratio of V: Al: C of 4:1.5:2.7 was sintered in an atmosphere furnace at 1,600°C and held for 2 h under flowing argon gas. Then, 2 g V_4_AlC_3_ (MAX) powders (with a particle size <38 μm) were slowly added into the 20 mL HF (49 wt%) solution. V_4_C_3_T_*x*_ MXene was thus prepared by etching in an oil bath at 55°C for a duration of 5 days. The etched powders were washed several times with ultrapure (UP) water, and centrifuged at 3,500 rpm for 180 s each time until the pH value of the supernatant was above 6. After that, the collected clay-like precipitates were put in the vacuum drying oven at 60°C for 24 h, namely, multi-layered V_4_C_3_T_*x*_ MXene. Secondly, 500 mg multi-layered V_4_C_3_T_*x*_ MXene powders were intercalated by 5 mL TBAOH, which was stirred at room temperature for 48 h. Followed that the resulted colloidal suspension was washed using ultrapure (UP) water, and centrifuged at 3,500 rpm for 180 s to separate the intercalated powders from the TBAOH liquid. After the decantation of the supernatant containing TBAOH, the residue and the mixture were dispersed into 100 mL UP water, deoxidized using the vacuum degassing device, ultrasonicated in the water bath for 1 h, and centrifuged at 3,500 rpm for 1 h. The final supernatant was collected and filtered on the mixed cellulose esters (MCE) membrane under a vacuum-assisted condition. After being dried in a vacuum oven for 12 h, the flexible V_4_C_3_T_*x*_ MXene film was easily peeled off the MCE membrane.

**FIGURE 1:**
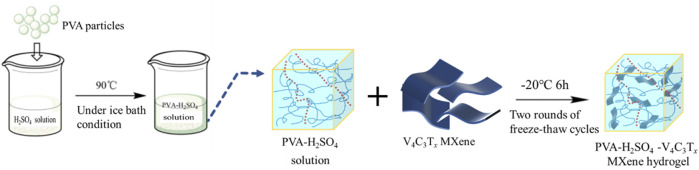
Preparation process of PVA-H_2_SO_4_-V_4_C_3_T_*x*_ MXene hydrogel electrolytes.

Subsequently, the preparation for the PVA-H_2_SO_4_-V_4_C_3_T_*x*_ MXene hydrogel electrolyte is shown in Figure 1, using a cyclic freeze-thawing for physical cross-linking coupled with a mechanical blending method. The details are as follows: (1) 4 g of polyvinyl alcohol (PVA) were combined with 28 mL of 3 M H_2_SO_4_ solution and 12 mL of ultrapure water in a 50 mL beaker. The mixture was then stirred and heated at 90°C for approximately 60 min until the PVA particles were completely dissolved under an ice bath condition; (2) After the PVA-H_2_SO_4_ solution was fully dissolved and cooled to room temperature, the solution was degassed to remove any bubbles. The solution was then divided into four equal parts and added back to the beaker. Then, 0 mg, 30 mg, 60 mg, and 90 mg of V_4_C_3_T_*x*_ MXene were added to each beaker while stirring. The mixture was stirred for 1 h to ensure uniform distribution and then cast into glass petri dishes with a diameter of 9 cm, labelled as PVA-H_2_SO_4_, PVA- H_2_SO_4_-413 MXene-30, PVA- H_2_SO_4_-413 MXene-60, and PVA- H_2_SO_4_-413 MXene-90; (3) The glass petri dishes containing the solution were covered with plastic wrap and placed in the refrigerator at −20°C for 6 h of freezing; (4) After freezing was over, the glass petri dishes were taken out and allowed to thaw at room temperature for 6 h until fully thawed; (5) The thawed samples were then placed back again in the refrigerator at −20°C for 6 h of freezing; (6) After freezing was completed, the samples were taken out and allowed to thaw at room temperature, thus the V_4_C_3_T_*x*_ MXene/polyvinyl alcohol hydrogel electrolytes were obtained.

### 2.2 Physical characterizations

The X-ray diffraction (XRD) patterns of the as-prepared samples were characterized by Rigaku D/max 2,200 pc diffractometer with Cu Kα radiation (λ = 1.5406 Å) in a 2θ angular range of 5°–80°. The morphological properties of the samples were observed by field-emission scanning electron microscopy (FE-SEM, Hitachi S-4800 and FEI company Tecnai G220 S-twin). Contact angles of the samples were measured by using the contact angle measurement instrument (DSA100S, KRUSS, Germany).

### 2.3 Electrochemical measurements

The electrochemical performances for the flexible solid-state supercapacitors were tested by a CHI 660E electrochemical workstation. The flexible solid-state symmetric supercapacitor was assembled using PET as the flexible substrate, copper foil as the conductive wire, and V_4_C_3_T_*x*_ MXene film as the positive and negative electrodes. The PVA-H_2_SO_4_, PVA-H_2_SO_4_-V_4_C_3_T_*x*_ with different contents of V_4_C_3_T_*x*_ MXene (PVA-H_2_SO_4_-413MXene-30, PVA-H_2_SO_4_-413MXene-60, and PVA-H_2_SO_4_-413MXene-90) hydrogels were used as the solid-state electrolytes, respectively. The electrochemical behaviours of the electrodes were explored by cyclic voltammetry (CV), galvanostatic charge-discharge (GCD), electrochemical impedance spectroscopy (EIS) measurements. In addition, the Landian CT2001A tester was used to evaluate the long cycling stability of the electrode material.

### 2.4 Calculation

Gravimetric capacitance (*C*_*g*_, F g^−1^) for the electrodes of flexible solid-state supercapacitors calculated from the GCD curves is given by the following formula:

$$C_{g}=4\;I\;\Delta t\;/\left(m\;\Delta V\right)$$

where *I* (A) is the constant discharging current of the GCD curve, *Δt* (V) is the discharging time of the GCD curve, *ΔV* (V) is the potential window, and *m* (g) is the total mass of electrodes for the flexible solid-state supercapacitor.

Gravimetric capacitance (*C*_*g*0_, F g^−1^) for the flexible solid-state supercapacitors can be obtained by the formula:

$$C_{g0}=1/4\;C_{g}$$

Energy density (*E*) and power density (*P*) of the flexible solid-state supercapacitor can be calculated via the following equations:

$$E=1/2\;C_{g0}\;\Delta V_{0}^{2}$$

$$P=3600\;E/\Delta t_{0}$$

where *ΔV*_0_ (V) is the voltage window and *Δt*_*0*_ is the time for a sweep segment for the flexible solid-state supercapacitor.

## 3 Results and discussion

**FIGURE 2:**
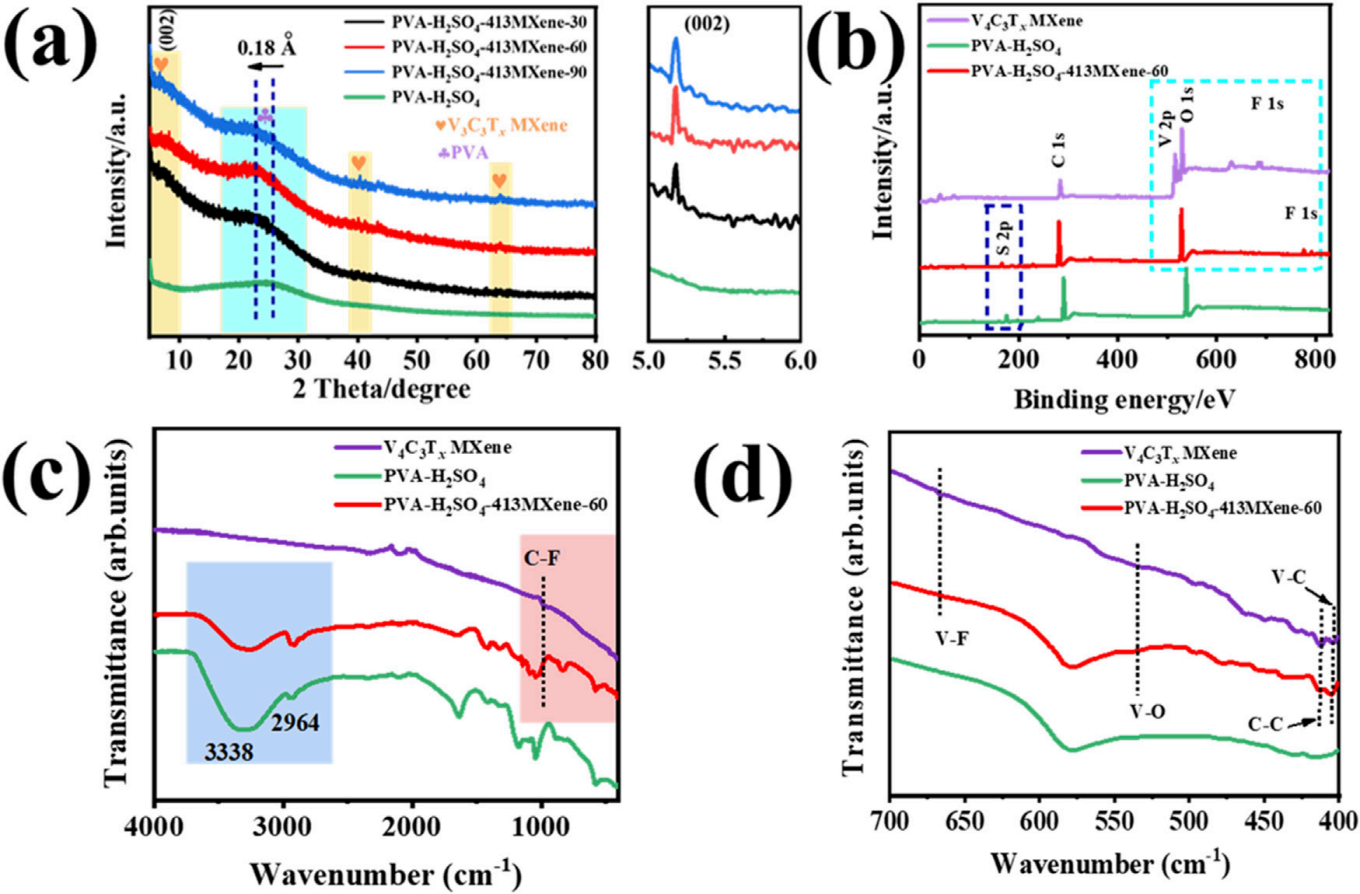
**(A)** XRD patterns of the hydrogel electrolyte samples for PVA-H_2_SO_4_, PVA-H_2_SO_4_-V_4_C_3_T_*x*_ with different contents of V_4_C_3_T_*x*_ MXene and the enlarged (002) peak. **(B)** XPS survey spectra, **(C)** IR spectra and **(D)** enlarged part of IR spectra for the V_4_C_3_T_*x*_ MXene, PVA-H_2_SO_4_, and PVA-H_2_SO_4_-413MXene-60 hydrogel electrolyte.

Figure 2 shows the X-ray diffraction (XRD) patterns of hydrogel electrolytes for PVA- H_2_SO_4_, and PVA- H_2_SO_4_-V_4_C_3_T_*x*_ with different contents of V_4_C_3_T_*x*_ MXene. It can be seen that all hydrogel electrolyte samples exhibit a broad and diffuse peak in the range of 2θ = 18°–30°, which is attributed to the characteristic peak of an amorphous structure for PVA (indicated by the blue region in the Figure 2A). This peak lacks of the sharp and well-defined nature typically seen in crystalline materials, reflecting the disordered and random arrangement of the polymer chains in the PVA matrix. Compared to the PVA-H_2_SO_4_ hydrogel electrolyte, the PVA-H_2_SO_4_-V_4_C_3_T_*x*_ MXene hydrogel electrolytes all show characteristic diffraction peaks corresponding to the preferential orientation (002) of V_4_C_3_T_*x*_ MXene (as indicated by the yellow area in the Figure 2A) (Bin et al., 2022; Bin et al., 2021), confirming the successful incorporation of V_4_C_3_T_*x*_ MXene into the PVA-H_2_SO_4_-V_4_C_3_T_*x*_ MXene hydrogel electrolytes. Besides, the characteristic peaks of PVA in PVA-H_2_SO_4_-V_4_C_3_T_*x*_ MXene hydrogel electrolytes are shifted towards a smaller 2θ angle, indicating an expansion of the interlayer spacing by 0.18 Å (calculated by Bragg’s law). This shift is likely attributed to the formation of more ordered structures in the amorphous regions induced by the incorporation of V_4_C_3_T_*x*_ MXene, or due to the influence of V_4_C_3_T_*x*_ MXene on the local structure of the amorphous regions, resulting in an increase in the average interplanar spacing and a leftward shift in the diffraction angle.

However, the intensity of the characteristic diffraction peak corresponding to V_4_C_3_T_*x*_ MXene is relatively weak, which might be due to the dynamic disorder of the MXene sheets as they move with the polymer chains, thereby reducing long-range orderliness and leading to a decrease in the intensity of the diffraction peak.

Furthermore, as shown in Figures 2B–D, the X-ray Photoelectron Spectroscopy (XPS) survey spectra and Infrared (IR) Spectroscopy for the V_4_C_3_T_*x*_ MXene, PVA-H_2_SO_4_, and PVA-H_2_SO_4_-413MXene-60 hydrogel electrolyte were tested. There are characteristic elements (V, C, O, F) and peaks (corresponding to V-C, C-C, V-O, V-F, and C-F stretching) for V_4_C_3_T_*x*_ MXene can be found from the PVA-H_2_SO_4_-413MXene-60 hydrogel electrolyte (Parker et al., 2024). It suggests that V_4_C_3_T_*x*_ MXene was successfully introduced into PVA molecular chains.

**FIGURE 3:**
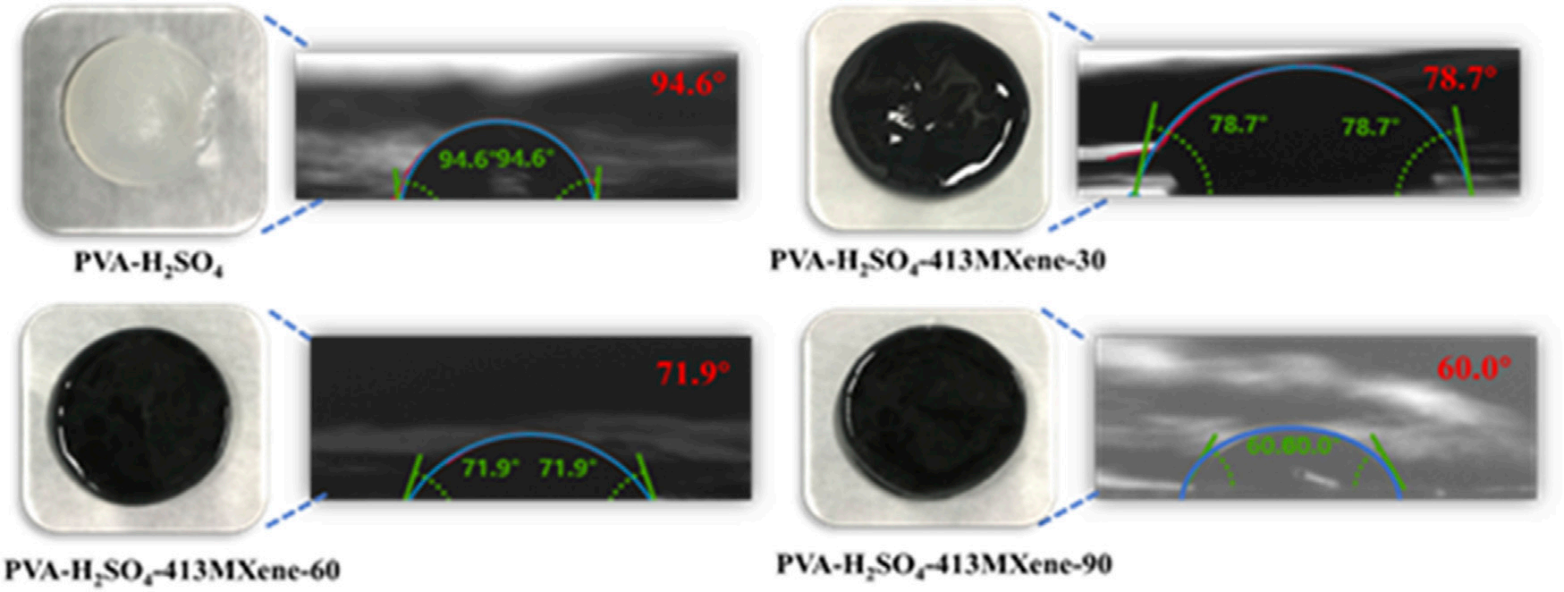
Photographs of hydrogel electrolytes for PVA-H_2_SO_4_, PVA-H_2_SO_4_-V_4_C_3_T_*x*_ MXene with different contents of V_4_C_3_T_*x*_ MXene, and contact angle measurement images.

Contact angle measurements were utilized to quantify the angle formed by a liquid droplet when it comes into contact with a solid substrate. This angle is a result of the equilibrium among the interfacial tension at the liquid-solid interface, the interfacial tension at the liquid-air interface, and the surface energy of the solid. Contact angle measurements are commonly performed using a camera in conjunction with specialized software. Here, a known volume of liquid is dispensed on the solid surface, and the camera captures the shape of the droplet. Subsequently, the software analyzes the image to determine the contact angle. The contact angle is helpful for characterizing the wettability of the solid surface, with angles less than 90° indicating hydrophilicity, angles greater than 90° indicating hydrophobicity, and angles greater than 150° indicating super hydrophobicity. As shown in Figure 3, the contact angle of the PVA- H_2_SO_4_ hydrogel is 94.6°, while the contact angles for the PVA-H_2_SO_4_-413MXene-30, PVA-H_2_SO_4_-413MXene-60 and PVA-H_2_SO_4_-413MXene-90 hydrogels are 78.7°, 71.9° and 60.0°, respectively. As the contents of V_4_C_3_T_*x*_ MXene increases, the contact angles for the hydrogel electrolytes decrease, indicating a transition towards greater hydrophilicity. This is attributed to the formation of hydrogen bonds between the hydroxyl groups (-OH) on the surface of MXene and the hydroxyl groups (-OH) on the PVA, which enhances the interfacial wettability of the hydrogel electrolytes.

**FIGURE 4:**
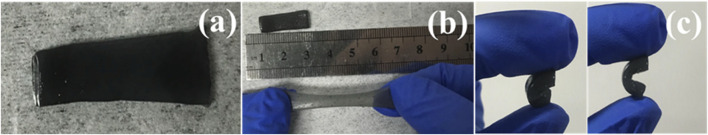
Photographs of the PVA-H_2_SO_4_-413MXene-60 hydrogel electrolyte: **(A)** Surface, **(B)** Stretching, and **(C)** Compression.

Figure 4A displays the optical microscopy images for the optimal PVA-H_2_SO_4_-413MXene-60 hydrogel electrolyte, revealing a smooth surface morphology. The mechanical properties for the optimal PVA-H_2_SO_4_-413MXene-60 hydrogel electrolyte were assessed. As shown in Figure 4B, it is evident that the PVA-H_2_SO_4_-413MXene-60 hydrogel electrolyte can be elastically stretched to more than twice its original length and rapidly returns to its original shape. Furthermore, the PVA-H_2_SO_4_-413MXene-60 hydrogel electrolyte can be easily compressed multiple times and rapidly rebounds, as seen in Figure 4C. These results indicate that the as-prepared PVA-H_2_SO_4_-413 MXene-60 hydrogel electrolyte possesses good tensile and compressive resistance, as well as superior flexibility.

**FIGURE 5:**
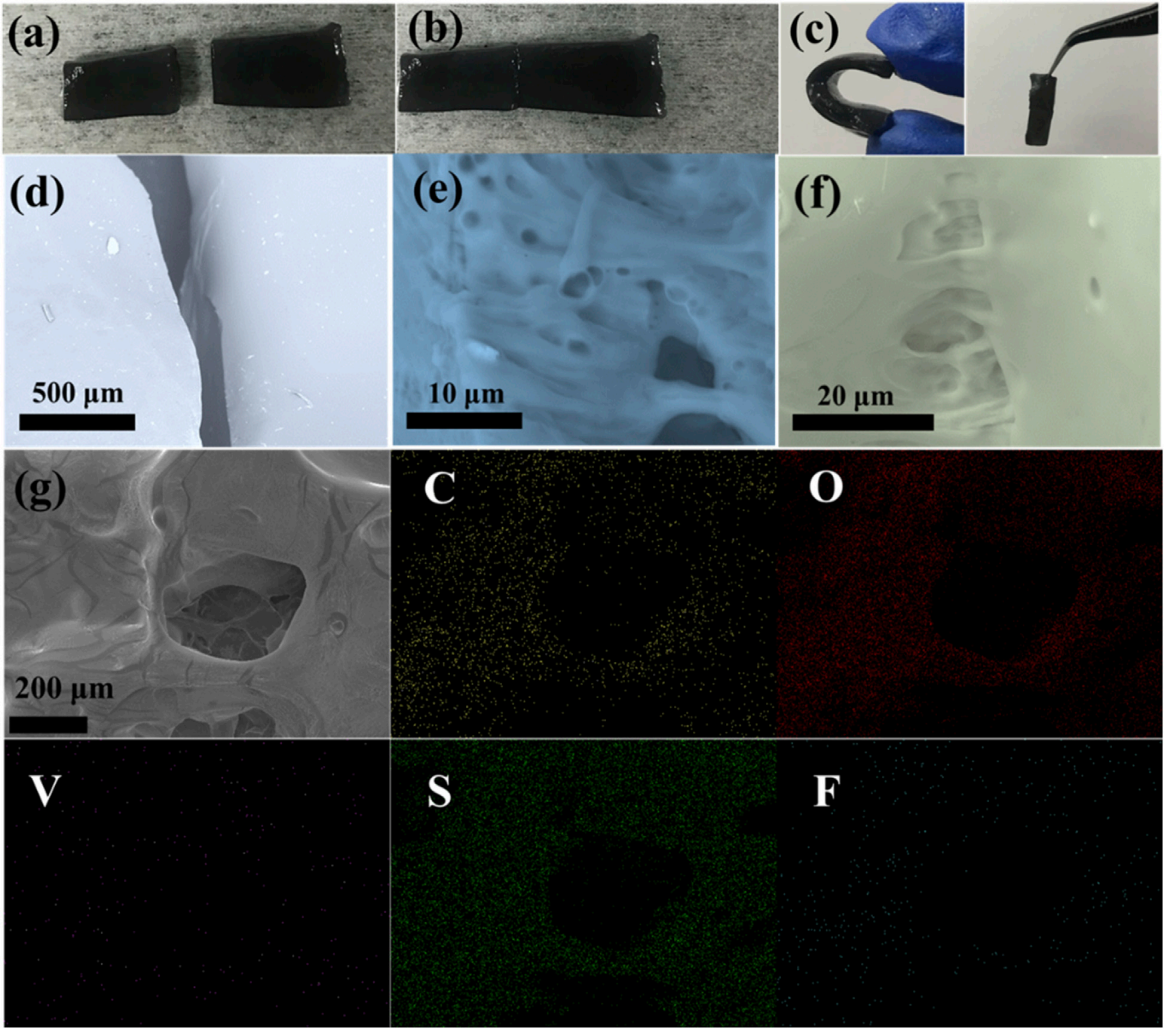
Photographs of cutting **(A)**, self-healing surface **(B)**, and compression and vertical suspension **(C)**, SEM images of the corresponding cutting **(D)**, the internal structure re-crosslinked **(E)**, the surface for the self-healing joint **(F)**, SEM image and the corresponding EDS elemental mapping images **(G)** for the PVA-H_2_SO_4_-413MXene-60 hydrogel electrolyte.

Furthermore, the optimal PVA-H_2_SO_4_-413MXene-60 hydrogel electrolyte also exhibits an excellent self-healing property upon physical damage. The PVA-H_2_SO_4_-413MXene-60 hydrogel electrolyte is cut into two parts as seen in Figure 5A, with the corresponding scanning electron microscopy (SEM) images shown in Figure 5D. Subsequently, the two separated parts can be naturally contacted together at room temperature. Figure 5B shows that the separated parts can easily reintegrate into a single entity after 30 min. The SEM morphology images for the hydrogel electrolyte at the partially connected areas (Figures 5E, F) reveal its three-dimensional porous structure, as well as show the internal structure re-crosslinked via hydrogen bonding. The Figure 5G shows the SEM image for the PVA-H_2_SO_4_-413MXene-60 hydrogel electrolyte, and the corresponding EDS elemental mapping images, including C, O, V, S, and F elements, which is consistent with the analysis results from XRD, XPS, and IR above. The hydroxyl groups (-OH) for the functional groups on the surface of V_4_C_3_T_*x*_ MXene form more hydrogen bonds with the hydroxyl groups (-OH) on the PVA molecules, which facilitates the rapid self-healing of the hydrogel electrolyte. Moreover, the unique three-dimensional structure for hydrogel electrolyte can promote ion transport, thereby enhancing its ionic conductivity. The mechanical property for the self-healing hydrogel electrolyte was further tested. As shown in Figure 5C, the self-healing hydrogel electrolyte is not deformed after being compressed again. Especially, the self-healing joint does not fall off when suspended vertically, and is still firmly adhered to together, which shows its good bending resistance.

To further investigate the electrochemical performance of hydrogel electrolytes for PVA-H_2_SO_4_, PVA-H_2_SO_4_-V_4_C_3_T_*x*_ with different contents of V_4_C_3_T_*x*_ MXene in flexible all-solid-state supercapacitors, the flexible solid-state symmetric supercapacitor was assembled using PET as the flexible substrate, copper foil as the conductive wire, and V_4_C_3_T_*x*_ MXene as the positive and negative electrodes. The electrochemical properties of flexible solid-state symmetric supercapacitors constructed using various hydrogel electrolytes were individually evaluated.

**FIGURE 6:**
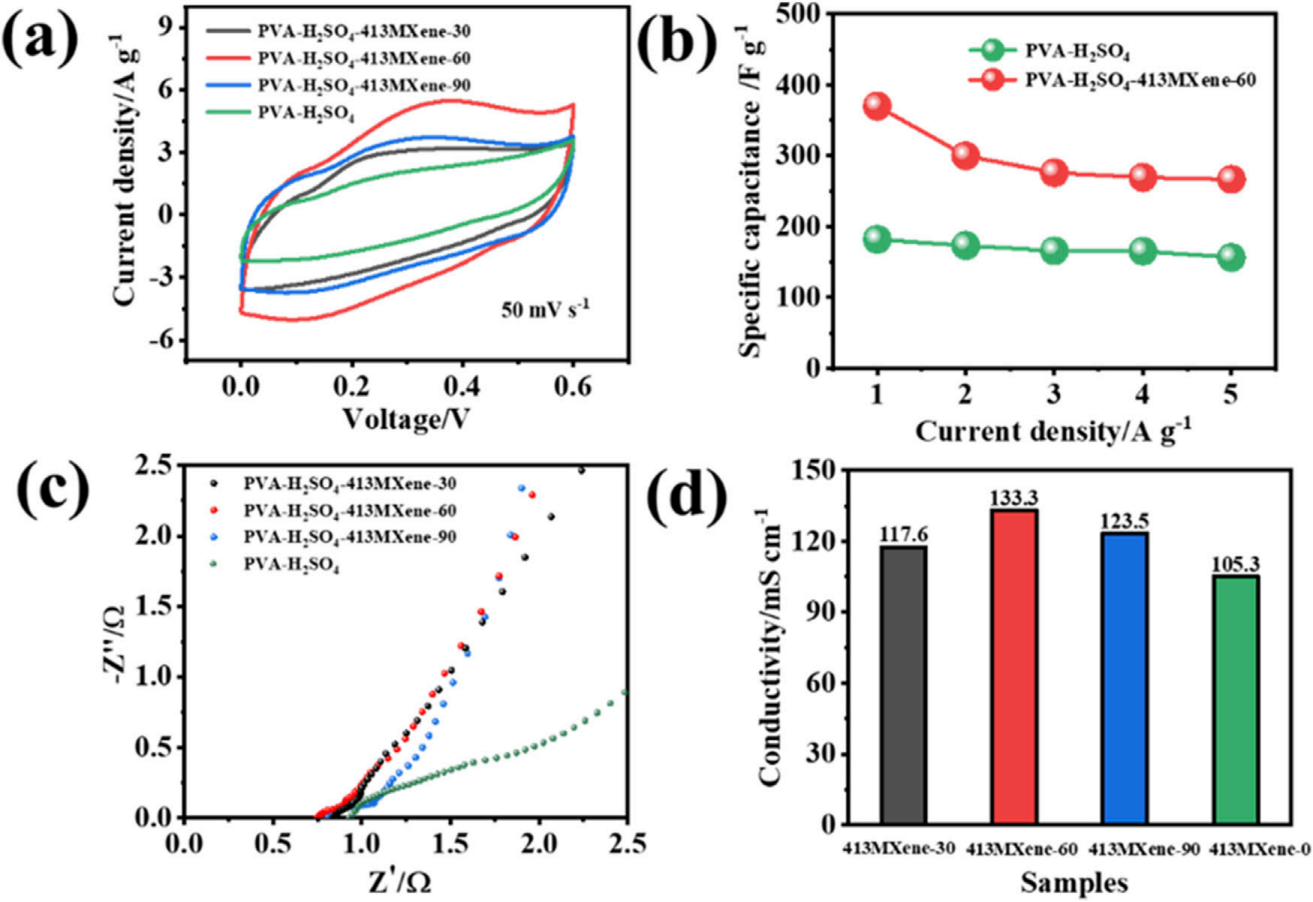
Electrochemical performance for the flexible solid-state symmetric supercapacitors: **(A)** CV curves, **(B)** capacitance values at different current densities, **(C)** electrochemical impedance spectroscopy (EIS), and **(D)** ionic conductivities for different hydrogel electrolytes.

Figure 6A shows the cyclic voltammetry (CV) tests for the hydrogel electrolytes at a scan rate of 50 mV s^-1^. It can be observed that the areas of CV curves for the PVA-H_2_SO_4_-V_4_C_3_T_*x*_ MXene hydrogel electrolytes are all larger than that of the PVA-H_2_SO_4_ hydrogel electrolyte, indicating that the PVA-H_2_SO_4_-V_4_C_3_T_*x*_ MXene hydrogel electrolytes are beneficial for the capacitive performance of the flexible solid-state supercapacitor. Obviously, the PVA-H_2_SO_4_-413MXene-60 hydrogel electrolyte has the largest area, which suggests that it has the highest capacitance value (370 F g^−1^) and thus is the optimal hydrogel electrolyte. Figure 6B presents the capacitance values for the PVA-H_2_SO_4_ and PVA-H_2_SO_4_-413MXene-60 hydrogel electrolytes at different current densities. At a current density of 1 A g^−1^, the electrode of the flexible device with PVA-H_2_SO_4_ as the solid-state electrolyte has a capacitance of 182 F g^−1^, while the electrode of the flexible device with PVA-H_2_SO_4_-413MXene-60 as the solid-state electrolyte has a capacitance of 370 F g^−1^, which is twice for that of the electrode for PVA-H_2_SO_4_ as the solid-state electrolyte. The enhancement mechanism of V_4_C_3_T_*x*_ MXene on polyvinyl alcohol containing sulfuric acid (PVA-H_2_SO_4_) electrolytes may be attributed to the formation of hydrogen bonds between the hydroxyl groups on the surface of V_4_C_3_T_*x*_ MXene and the PVA chains, the interaction between sulfate ions (SO_4_^2−^) and the metal sites on the V_4_C_3_T_*x*_ MXene, as well as the van der Waals forces between the V_4_C_3_T_*x*_ MXene nanosheets and PVA molecules, which thus improves the interfacial hydrophilicity for the hydrogel electrolytes, enhances the interfacial contact between the electrodes and the solid-state electrolytes, reduces interfacial internal resistance, as well as boosts charge storage efficiency. Besides, the large specific surface area and abundant surface functional groups of V_4_C_3_T_*x*_ MXene provide more active sites for electrochemical reactions, increase the contact area between the electrolyte ions and the electrode materials, and thus enhance the capacitance. Moreover, the high electrical conductivity for V_4_C_3_T_*x*_ MXene facilitates ion transport in the solid-state electrolytes, reduces resistance, and increases ionic conductivity, thereby enhancing the overall capacitance for the flexible solid-state supercapacitors.

Furthermore, the ionic conductivities of the hydrogel electrolytes were calculated by analyzing the electrochemical impedance spectroscopy (EIS), using the formula δ = d/(R × S), where δ is the ionic conductivity for the hydrogel electrolyte (mS cm^−1^), d is the distance between the two current collectors (cm), R is the resistance of the hydrogel electrolyte (Ω), and S is the contact area between the current collector and the electrolyte (cm^2^). As shown in Figures 6C, D, the ionic conductivities for PVA-H_2_SO_4_, PVA-H_2_SO_4_-413MXene-30, PVA-H_2_SO_4_-413MXene-60, and PVA-H_2_SO_4_-413MXene-90 are 105.3 mS cm^−1^, 117.6 mS cm^−1^, 133.3 mS cm^−1^, and 123.5 mS cm^−1^, respectively. Also, the PVA-H_2_SO_4_-413MXene-60 hydrogel electrolyte with the optimal content for V_4_C_3_T_*x*_ MXene has the highest ionic conductivity.

**FIGURE 7:**
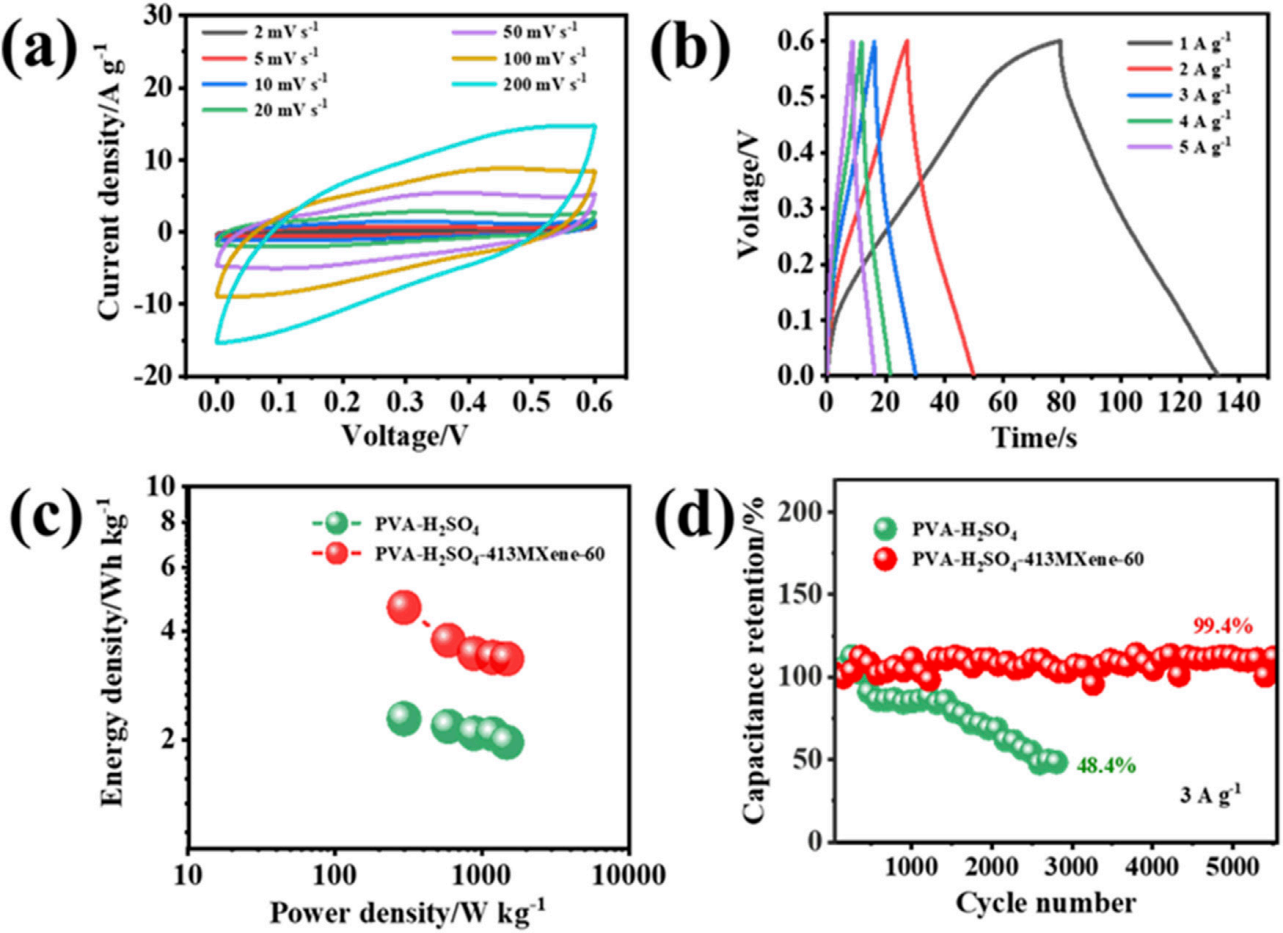
Electrochemical performance for the flexible solid-state supercapacitors with PVA-H_2_SO_4_ hydrogel and PVA-H_2_SO_4_-413MXene-60 hydrogel as the solid-state electrolytes: **(A)** CV curves, **(B)** GCD curves, **(C)** Ragone plots, **(D)** Long-term cycling performance testing.

The electrochemical properties of the flexible solid-state symmetric device assembled with the PVA-H_2_SO_4_-413MXene-60 hydrogel electrolyte were examined. As depicted in Figures 7A, B, even at a high scan rate of 200 mV s^−1^, the CV curves do not appear severe distortion and maintain a quasi-rectangular shape, while the GCD curves also exhibit good symmetry, which has as a quasi-triangular shape, indicating that the flexible solid-state supercapacitor has good reversibility. The Ragone plots in Figure 7C illustrate that flexible device with PVA-H_2_SO_4_-413MXene-60 hydrogel as the solid-state electrolyte achieves an energy density of 4.6 Wh kg^-1^ at a power density of 300 W kg^−1^, while the flexible device with PVA-H_2_SO_4_ hydrogel as the solid-state electrolyte only has an energy density of 2.3 Wh kg^−1^. Moreover, the flexible supercapacitor with PVA-H_2_SO_4_-413MXene-60 hydrogel as the solid-state electrolyte demonstrates superior cycling stability (Figure 7D), that is, at a current density of 3 A g^−1^, the device retains 99.4% of its original capacitance after 5,500 cycles, whereas the flexible supercapacitor with PVA-H_2_SO_4_ hydrogel as the solid-state electrolyte degrades to 48.4% of its original capacitance after only 2,800 cycles. The enhanced cycling performance for the PVA-H_2_SO_4_-413MXene-60 hydrogel electrolyte is attributed to the incorporation of V_4_C_3_T_*x*_ MXene, which results in a hydrogel electrolyte with high ionic conductivity and good interfacial compatibility.

**FIGURE 8:**
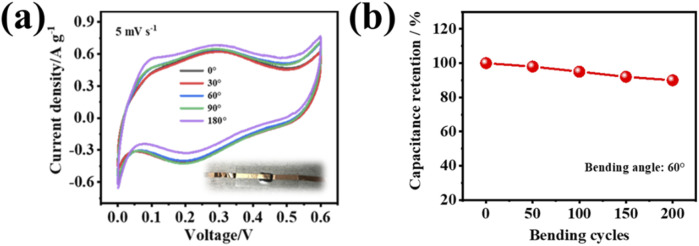
**(A)** The CV curves for the flexible solid-state supercapacitor based on PVA-H_2_SO_4_-413MXene-60 hydrogel electrolyte at different bending angles at a scan rate of 5 mV s^−1^, with an inset showing a photograph of the fabricated flexible solid-state supercapacitor. **(B)** The capacitance retention with various bending cycles of PVA-H_2_SO_4_-413MXene-60 hydrogel electrolyte.

To evaluate the bending resistance of the PVA-H_2_SO_4_-413MXene-60 hydrogel electrolyte, a flexible supercapacitor with PVA-H_2_SO_4_-V_4_C_3_T_*x*_ MXene-60 hydrogel as the solid-state electrolyte was subjected to cyclic voltammetry (CV) testing at bending angles of 0°, 30°, 60°, 90°, and 180°. As seen in Figure 8A, the CV curves for different bending angles at a scan rate of 5 mV s^−1^ show similar areas, indicating that their capacitance almost have no degradation, and bending angles have little impact on the specific capacitance for the supercapacitor. After 200 bending cycles (Figure 8B), it still maintained 90% capacitance retention, indicating that the hydrogel electrolyte has good durability. It also suggests that the flexible solid-state supercapacitor based PVA-H_2_SO_4_-413MXene-60 hydrogel electrolyte possesses good flexibility and bending resistance performance.

## 4 Conclusion

To solve the low ionic conductivity, large interfacial resistance and poor cycling stability for hydrogel electrolytes, the high-conductivity V_4_C_3_T_*x*_ MXene-enhanced polyvinyl alcohol hydrogel electrolytes (PVA-H_2_SO_4_-V_4_C_3_T_*x*_ MXene) have been successful prepared by cyclic freeze-thawing for physical cross-linking coupled with a mechanical blending method. The PVA is used as the polymer matrix, while sulfuric acid (H_2_SO_4_) is used as the proton source, and V_4_C_3_T_*x*_ MXene is used as the inorganic filler embedded into the PVA-H_2_SO_4_ hydrogel electrolyte via MXene bonding transport network. Compared to the pure PVA-H_2_SO_4_ hydrogel electrolyte (105.3 mS cm^−1^, 48.4%@2,800 cycles), the optimal PVA-H_2_SO_4_-V_4_C_3_T_*x*_ MXene hydrogel electrolyte demonstrates high ionic conductivity (133.3 mS cm^−1^) and commendable long-cycle stability for the flexible solid-state supercapacitors (99.4%@5,500 cycles), and exhibits favorable mechanical flexibility and self-healing capability. Besides, the electrode of the flexible solid-state supercapacitor with the optimal PVA-H_2_SO_4_-V_4_C_3_T_*x*_ MXene as the solid-state electrolyte has a capacitance of 370 F g^−1^ with almost no degradation in capacitance even under bending from 0° to 180°. The corresponding energy density for flexible device is 4.6 Wh kg^−1^, which is twice for that of PVA-H_2_SO_4_ hydrogel as the solid-state electrolyte. The enhancement mechanism of V_4_C_3_T_*x*_ MXene on polyvinyl alcohol containing sulfuric acid PVA-H_2_SO_4_ hydrogel may be attributed to the formation of hydrogen bonds between the hydroxyl groups on the surface of MXene and the PVA chains, the interaction between sulfate ions (SO_4_^2−^) and the metal sites on the MXene, as well as the van der Waals forces between the MXene nanosheets and PVA molecules, thus strengthening the interfacial compatibility, the ionic conductivity, and the structural stability for the hydrogel electrolytes. The improvement for hydrogel electrolyte property is great importance for the development of high-performance flexible solid-state supercapacitors.

## Data Availability

The original contributions presented in the study are included in the article/Supplementary Material, further inquiries can be directed to the corresponding author.
