# Supplementary material for: Highly conductive V4C3T x MXene-enhanced polyvinyl alcohol hydrogel electrolytes for flexible all-solid-state supercapacitors
Source: Front Chem. 2024 Oct 9;12:1482072. doi: 10.3389/fchem.2024.1482072 (PMC11496089; doi:10.3389/fchem.2024.1482072)
Supplement: Supplementary file 1 [file Table1.docx]

**Supporting Information**

**Highly Conductive V_4_C_3_T*_x_* MXene-Enhanced** **Polyvinyl Alcohol Hydrogel Electrolytes for Flexible All-Solid-State Supercapacitors**

**Xiaoqing Bin,** **Minhao Sheng, and** **Wenxiu Que** **^*^**

Electronic Materials Research Laboratory, Key Laboratory of the Ministry of Education, International Center for Dielectric Research, Shaanxi Engineering Research Center of Advanced Energy Materials and Devices, School of Electronic Science and Engineering, Xi’an Jiaotong University, Xi’an 710049, People’s Republic of China

*** Correspondence:**Corresponding Author: Wenxiu Que
wxque@mail.xjtu.edu.cn

The self-discharge rates of the PVA-H_2_SO_4_-413MXene-60 hydrogel electrolyte were tested at room temperature. The detailed performance data accordingly are following:

Table R1 the self-discharge performance of the PVA-H_2_SO_4_-413MXene-60 hydrogel electrolyte

| Time (h) | 0 | 6 | 12 | 18 | 24 |  |
| --- | --- | --- | --- | --- | --- | --- |
| Voltage (V) | 0.60 | 0.56 | 0.52 | 0.5 | 0.49 | |

Self-discharge rate for 6 h: [(0.60 V - 0.56 V)/0.6 V]*100% = 6.7%

Self-discharge rate for 12 h: [(0.60 V - 0.52V)/0.6 V]*100% = 13.3%

Self-discharge rate for 18 h: [(0.60 V - 0.5 V)/0.6 V]*100% = 16.7%

Self-discharge rate for 24 h: [(0.60 V - 0.49 V)/0.6 V]*100% = 18.3%
